# Nociceptor Deletion of Tsc2 Enhances Axon Regeneration by Inducing a Conditioning Injury Response in Dorsal Root Ganglia

**DOI:** 10.1523/ENEURO.0168-19.2019

**Published:** 2019-06-25

**Authors:** Dan Carlin, Alexandra E. Halevi, Eric E. Ewan, Amy M. Moore, Valeria Cavalli

**Affiliations:** 1 Department of Neuroscience, Washington University School of Medicine, St. Louis, MO 63110; 2 Division of Plastic and Reconstructive Surgery, Washington University School of Medicine, St. Louis, MO 63110; 3 Department of Neuroscience, Center of Regenerative Medicine, Hope Center for Neurological Disorders, Washington University School of Medicine, St. Louis, MO 63110

**Keywords:** axon regeneration, dorsal root ganglion, macrophages, mTORC1, nociceptor, Tsc2

## Abstract

Neurons of the PNS are able to regenerate injured axons, a process requiring significant cellular resources to establish and maintain long-distance growth. Genetic activation of mTORC1, a potent regulator of cellular metabolism and protein translation, improves axon regeneration of peripheral neurons by an unresolved mechanism. To gain insight into this process, we activated mTORC1 signaling in mouse nociceptors via genetic deletion of its negative regulator Tsc2. Perinatal deletion of Tsc2 in nociceptors enhanced initial axon growth after sciatic nerve crush, however by 3 d post-injury axon elongation rate became similar to controls. mTORC1 inhibition prior to nerve injury was required to suppress the enhanced axon growth. Gene expression analysis in purified nociceptors revealed that Tsc2-deficient nociceptors had increased activity of regeneration-associated transcription factors (RATFs), including cJun and Atf3, in the absence of injury. Additionally, nociceptor deletion of Tsc2 activated satellite glial cells and macrophages in the dorsal root ganglia (DRG) in a similar manner to nerve injury. Surprisingly, these changes improved axon length but not percentage of initiating axons in dissociated cultures. The pro-regenerative environment in naïve DRG was recapitulated by AAV8-mediated deletion of Tsc2 in adult mice, suggesting that this phenotype does not result from a developmental effect. Consistently, AAV8-mediated Tsc2 deletion did not improve behavioral recovery after a sciatic nerve crush injury despite initially enhanced axon growth. Together, these data show that neuronal mTORC1 activation induces an incomplete pro-regenerative environment in the DRG that improves initial but not later axon growth after nerve injury.

## Significance Statement

Long-distance axon regeneration poses a significant hurdle to recovery following nervous system injury. Increased mTORC1 signaling improves axon regeneration, however the underlying mechanisms are incompletely understood. We activated neuronal mTORC1 signaling by genetically deleting Tsc2 in Nav1.8-positive neurons perinatally or by AAV8-mediated viral infection in adult mice and observed improved short- but not long-term axon regeneration after sciatic nerve injury. We suggest that Tsc2 deletion promotes initial but not later peripheral axon regeneration by upregulating expression of neuronal pro-regenerative genes and activating non-neuronal responses in the surrounding environment. Activating mTORC1 signaling in peripheral neurons may provide therapeutic benefit in circumstances with poor initial growth such as after spinal cord injury to the dorsal column or peripheral nerve repair.

## Introduction

Long-distance regeneration of injured axons poses an insurmountable challenge for adult neurons of the CNS, yet one that peripheral axons can overcome. Lack of regeneration after axon injury results in potentially devastating motor, cognitive, emotional and social deficits incurred from CNS insults such as stroke and spinal cord injury. In contrast, functional recovery can be achieved in the PNS after injury. However, long-term denervation of distal nerve and/or target tissue negatively impacts recovery (Gordon et al., 2011), which can occur in proximal injuries requiring very long-distance axon growth. In the regeneration-competent PNS, local protein synthesis in the injured axon is required to form a growth cone and promote initial growth of the axon (Bradke et al., 2012). Calcium and retrograde signaling from the injury site to the cell body establish a pro-regenerative gene expression program that is required to maintain long-distance axon growth (Mahar and Cavalli, 2018). In addition to the neuronal response, glia and immune cells provide trophic support and debris clearance (Huang et al., 2013; Christie et al., 2015; Krishnan et al., 2018; Jessen and Arthur-Farraj, 2019; Zigmond and Echevarria, 2019). Establishment of a full regeneration program in PNS is not evident for 3–7 d following injury, and as such, initial rate of axon elongation is notably less than rates at later time points (Smith and Skene, 1997; Lankford et al., 1998). Understanding a pathway’s role in initiation and/or elongation rate of regenerating axons is critical to evaluate its potential as a therapeutic target to improve functional recovery after PNS injury.

Activation of mTORC1 signaling through genetic deletion of negative regulators such as Pten, Tsc1, or Tsc2 improves axon regeneration in both the CNS and PNS (Park et al., 2008; Abe et al., 2010; Liu et al., 2010; Lee et al., 2014). mTORC1 signaling is a major effector of cellular growth through positive regulation of cellular metabolism and protein translation while also inhibiting protein turnover (Saxton and Sabatini, 2017). As such, mTORC1 may play a role in both initiation and elongation of axons. Initiation of CNS axon regeneration can be conferred by mTORC1 activation (Park et al., 2008; Liu et al., 2010); however, combining mTORC1 activation with dysregulation of other pathways including Stat3, B-raf, and c-Myc confer additional axon growth benefit over mTORC1 activation alone (Sun et al., 2011; O'Donovan et al., 2014; Belin et al., 2015). This suggests mTORC1 activation alone is not sufficient for CNS axons to achieve their full regenerative potential. The role of mTORC1 in peripheral axon regeneration is less clear. mTORC1 signaling in sensory axons is required for formation of a growth cone as well as retrograde survival signals after axon injury (Verma et al., 2005; Terenzio et al., 2018). While mTORC1 inhibition does not affect early axon regeneration *in vivo*, it does reduce axon growth of sensory neurons after a conditioning injury (Abe et al., 2010; Chen et al., 2016), suggesting mTORC1 may be required during later stages of axon regeneration. While genetic mTORC1 activation in sensory neurons enhances axon regeneration within 2 d after nerve injury (Abe et al., 2010), its effect on long-term regeneration and underlying mechanism remain to be resolved. As genetic mTORC1 activation in nociceptors does not induce baseline hypersensitivity (Carlin et al., 2018), this pathway may be of therapeutic relevance in promoting recovery after PNS injury. In the present study, we sought to evaluate the mechanism by which genetic mTORC1 activation improves PNS axon regeneration after nerve injury and to determine whether this mechanism can be recapitulated in adult mice.

## Materials and Methods

### Experimental design and statistical analyses

Full details for individual experiments are noted below. In general, analysis was performed by pooling near-equal numbers of male and female mice from at least two litters comparing homozygous *Tsc2* deletion to heterozygous littermate controls. Experimenters performing surgery and behavioral observations were blind to genotype. Image analysts were blind to both genotype and injury status. RNA sequencing (RNA-seq) analysis was performed with DESeq2 as described below. For all other experiments, GraphPad Prism software was used for statistical analysis with the appropriate tests, replicate numbers and statistical values cited in the extended figures. Statistical significance was defined by *p* < 0.05. All error bars denote SEM. Values of individual biological replicates are plotted for most experiments.

### Animals and procedures

All animal procedures and treatments were performed in accordance with the Washington University School of Medicine Institutional Animal Care and Use Committee’s regulations. Mice were housed in an AAALAC-accredited animal facility with 12/12 h light/dark cycles and *ad libitum* access to food and water. *Tsc2^fl/fl^* (floxed allele; RRID:MGI:3712786; Hernandez et al., 2007), *Tsc2^null/+^* (targeted null allele; RRID:MGI:2174787; Onda et al., 1999), *Nav1.8^Cre/+^* (also known as SNS-Cre, MGI:3042874; Agarwal et al., 2004), and *Rosa26-ZsGreen* (also known as Ai6(RCL-ZsGreen), RRID:IMSR_JAX:007906; Madisen et al., 2010) mice were described previously. For Tsc2 deletion mediated by Nav1.8-Cre, mice with genotype *Nav1.8^Cre/+^; Tsc2^null/+^* were crossed with *Tsc2^fl/fl^* mice. *Nav1.8^Cre/+^; Tsc2^null/fl^* mice are referred to as Tsc2 cKO mice. Littermate animals with genotypes *Tsc2^fl/+^*, *Tsc2^null/fl^*, and *Nav1.8^Cre/+^; Tsc2^fl/+^* were pooled as controls as they showed no phenotypic differences from each other. For AAV8-mediated deletion of Tsc2, mice with the genotypes *Tsc2^fl/+^*; *Rosa26-ZsGreen^GFP/GFP^* and *Tsc2^fl/fl^*; *Rosa26-ZsGreen^GFP/GFP^* were used as control and experimental (Tsc2 KO-AAV8) animals, respectively. Genotype was determined by PCR at weaning. Adult male and female mice aged 7–18 weeks were used for experiments.

For nerve injury experiments, mice were anesthetized with 2.5% isoflurane. Subcutaneous injection of 1 mg/kg buprenorphine SR-LAB (ZooPharm) was administered as an analgesic. The right thigh was shaved and disinfected with povidone-iodine solution (Ricca) and alcohol preps. The sciatic nerve was exposed and crushed with 10 s of full pressure from a #5 forceps (Fine Science Tools) or transected with scissors. For sham surgeries, the sciatic nerve was exposed but not crushed. For *in vivo* conditioning experiments, the second crush was performed >5 mm proximal to the initial crush site. The wound was closed with 6-0 Ethilon sutures and wound clips, which were removed at 7 d post-surgery for behavior experiments. For behavioral recovery experiments, mice were anesthetized via intraperitoneal injection of a 0.25 ml of 100 mg/ml ketamine and 0.5 mg/ml dexmedetomidine and resuscitated following surgery with 0.1 ml intraperitoneal Antisedan and 0.1 ml of 1 mg/kg solution of buprenorphine sustained release.

Rapamycin (LC Laboratories catalog #53123-88-9) was dissolved in ethanol diluted in 5% Tween 80, 5% PEG-400. Daily injections of vehicle or 5 mg/kg rapamycin were administered by subcutaneous injection.

For AAV8-mediated gene deletion, seven-week-old mice were anesthetized with 2.5% isoflurane and received intrathecal injections of AAV8-Cre-IRES-GFP under the CMV promoter according to previously reported methods (Njoo et al., 2014). Briefly, a 0.3-ml syringe with a 31-G needle was inserted between the groove of the L5 and L6 vertebrae, with the bevel of the needle facing in the rostral direction. After an observable tail flick indicative of needle entry into the intradural space, 5 μl of AAV8 was injected intrathecally, and the needle was left in place for 1 min before withdrawal. A second dose was administered 24 h later. Sciatic nerve crush surgery was performed three weeks after initial dose.

### Tissue processing and immunohistochemistry

Following euthanasia, mice were transcardially perfused with PBS followed by 4% paraformaldehyde in PBS (FD Neurotechnologies catalog #PF101). Sciatic nerve and/or L4 dorsal root ganglia (DRG) were isolated and immersed in 4% paraformaldehyde. Tissue was washed and cryoprotected in 30% sucrose. Frozen sections of 12 or 18 μm were obtained on a Leica CM1860 cryostat for nerve and DRG, respectively.

Immunostaining was performed as follows. Following a brief post-fixation in 4% paraformaldehyde and several washes in PBS with 0.1% Triton X-100 (PBSTx), sections were blocked using 5% donkey serum dissolved in PBSTx. Subsequently, sections were incubated overnight at 4°C in primary antibodies diluted in blocking reagent: Tsc2 (D93F12, 1:200; Cell Signaling catalog #4308, RRID:AB_10547134), SCG10/Stmn2 (1:1000; Novus catalog #NBP1-49461, RRID:AB_10011569), cJun (1:250; Cell Signaling catalog #9165, RRID:AB_2130165), Atf3 (1:250; Novus catalog #NBP1-85816, RRID:AB_11014863), Islet1 (1:500; R and D Systems catalog #AF1837, RRID:AB_2126324), Gfap (1:1000; Agilent catalog #Z0334, RRID:AB_10013382), and Iba1 (1:1000; Wako catalog #019-19741, RRID:AB_839504). *Griffonia simplicifolia* isolectin B4 (IB4) directly conjugated to Alexa Fluor 488 or Alexa Fluor 594 (1:250; Thermo Fisher Scientific catalog #I21411 and #I21413) was incubated with primary antibodies. Tubb3/βIII tubulin antibody (BioLegend catalog #802001, RRID:AB_291637) was directly conjugated to Alexa Fluor 594 or Alexa Fluor 647 using Apex labeling kit (Thermo Fisher Scientific) and incubated with primary antibodies at 1:200 dilution. Tissue was washed several times with PBSTx, incubated with fluorescent-conjugated secondary antibodies (1:500; Thermo Fisher Scientific) and DAPI (1:1000) diluted in blocking reagent, washed and mounted in ProLong Gold antifade mountant (Thermo Fisher Scientific). Images were taken with a Nikon TE-2000E compound microscope and Prior ProScan3 motorized stage, which automatically stitched images in Nikon Elements software. Images were analyzed using FIJI software (NIH).

### Image analysis

For sciatic nerve regeneration experiments, the crush site was determined visually in images by highest levels of SCG10 and confirmed by regional increased binding of IB4 (data not shown). A vertical line was drawn at the crush site, and the longest 10 axons were measured from this line and averaged. Three sections were averaged to establish the value of the longest axons for each biological replicate.

For cell counting experiments, total number of neurons as determined by the number of Tubb3-positive or Islet1-positive profiles as well as the number of marker-positive profiles were determined for each section. Only sections with >80 neurons were scored. The numbers of cells in each category for three sections were summed to generate a percentage of neurons expressing the marker(s) for each biological replicate.

To determine macrophage density, profiles double-positive for DAPI and Tubb3 were counted, and the area immediately surrounding the counted neurons was outlined. Images were thresholded for Iba1 immunoreactivity in a blinded manner and made binary. Iba1-positive area was determined in square pixels. The Iba1-positive area was summed for three sections and normalized to the total number of neurons in the regions of interest for each biological replicate. Simple area fraction was not used because of differences in neuronal cell size as a result of Tsc2 deletion (Carlin et al., 2018).

For satellite glial cell activation, profiles double-positive for DAPI and Tubb3 were counted. The percentage of those neurons that had Gfap-positive signal on at least two sides of the neuron cell body was determined and reported. Only sections with >80 neurons were scored. The numbers of cells in each category for three sections were summed to generate a percentage of neurons surrounded by Gfap for each biological replicate.

### Flow cytometry and RNA-seq analysis

Flow cytometry and gene expression analysis were characterized previously (Carlin et al., 2018). Uninjured control and Tsc2 cKO data from that study were reanalyzed for the present study. Briefly, L4 DRG from *Nav1.8^Cre/+^; Tsc2^fl/+^; Rosa26-ZsGreen^GFP/+^* (control) and *Nav1.8^Cre/+^; Tsc2^fl/null^; Rosa26-ZsGreen^GFP/+^* (Tsc2 cKO) contralateral and ipsilateral to a sciatic nerve crush were isolated 3 d after injury, dissociated as above, passed through a 70-μm cell strainer, resuspended in PBS with 2% fetal calf serum and subjected to flow cytometry. Cells were run through an 85-μm nozzle at 45 psi on a BD FACS Aria II machine.

A total of 100 L4 DRG cells were FACS-sorted for GFP signal into 9 μl Clontech lysis buffer with 5% RiboLock RNase Inhibitor for each sample. Three technical replicates of 100 cells each were sorted. All samples were submitted to the Genome Technology Access Center at Washington University School of Medicine for library preparation and sequencing. Libraries were prepared and sequenced separately for each technical replicate. Library preparation was performed using the SMARTer Ultra Low RNA kit for Illumina Sequencing (Clontech) per manufacturer’s protocol. cDNA was amplified for 13 cycles and then fragmented using a Covaris E220 sonicator using peak incident power 18, duty cycle 20%, cycles/burst 50, time 120 s at room temperature. cDNA was blunt ended, had an A base added to the 3’ ends, and then had Illumina sequencing adapters ligated to the ends. Ligated fragments were then amplified for 15 cycles using primers incorporating unique index tags. Fragments were sequenced on an Illumina HiSeq-3000 using single reads extending 50 bases. Samples were QC’d using FastQC, aligned to mm10 using STAR-align, and counted using HTseq-count. Technical replicates were collapsed in RStudio and differential expression determined using DESeq2. The threshold for differential expression was defined as adjusted *p* < 0.05 and log2 fold change >0.5 or <–0.5. The top differentially regulated Gene Ontology (GO) categories and upregulated transcription factor target genes were determined using Metacore software (Clarivate Analytics) with FDR threshold <0.05. oPOSSUM 3.0 was used for transcription factor binding site analysis of upregulated genes for each condition relative to uninjured control (Kwon et al., 2012). For transcription factor binding site analysis, JASPAR CORE profiles were used with all 29,347 genes in the oPOSSUM database as background genes. 5000 base-pairs of upstream and downstream sequence were analyzed.

RNA-seq FastQ and HTseq-count files were deposited at the NCBI GEO database (https://www.ncbi.nlm.nih.gov/geo/) under accession numbers GSE112499 (Carlin et al., 2018) and GSE125685 (present study).

### Western blotting

Adult L4/L5 DRG or sciatic nerves were isolated and manually homogenized in 2% sodium dodecyl sulfate in 60 mM Tris, pH 6.8 with protease and phosphatase inhibitors (Roche Applied Sciences). Protein concentration was determined by DC protein assay (Bio-Rad Laboratories) against bovine serum albumin standards. Twelve- to 15-μg total protein was loaded onto 4–12% Bis-Tris polyacrylamide gels (Invitrogen). Nitrocellulose membranes were blotted with antibodies directed against the following proteins: α-tubulin (1:20,000; Abcam catalog #ab18251, RRID:AB_2210057), cJun (1:1000; Cell Signaling catalog #9165, RRID:AB_2130165), phospho-S6 S240/244 (1:1000; Cell Signaling Technology catalog #5364, RRID:AB_10694233), S6 (1:1000; (Cell Signaling Technology catalog #2217, RRID:AB_331355), Atf3 (1:1000; Novus catalog #NBP1-85816, RRID:AB_11014863), phospho-Stat3 Y705 (1:1000; Cell Signaling Technology catalog #9131, RRID:AB_331586), Stat3 (1:1000; Cell Signaling Technology catalog #12640, RRID:AB_2629499), and rabbit IgG conjugated to horseradish peroxidase (1:10,000; Thermo Fisher catalog #656120). Initially, antibodies for phosphorylated isoforms were probed, membranes were stripped in 60 mM Tris-HCl, 2% sodium dodecyl sulfate, pH 6.8 at 50°C for 30 min, washed extensively, and then probed for total protein. Blots were developed with SuperSignal West Dura (ThermoFisher), imaged with a ChemiDoc MP imaging system and quantified with Image Lab 5.2.1 (Bio-Rad Laboratories). Volume intensity of markers was first normalized to α-tubulin. Log2 fold change of normalized values in relation to normalized uninjured control value was determined for each biological replicate.

### Quantitative PCR

Adult L3 (rapamycin experiments) or L4/L5 (injury experiments) DRG were isolated, lysed, and homogenized. Total RNA was extracted with PureLink RNA Mini kit according to manufacturer’s instructions (Thermo Fisher Scientific). RNA concentration was determined by NanoDrop 2000 (Thermo Fisher Scientific). Samples were reverse transcribed with High Capacity cDNA Reverse Transcription kit (Applied Biosystems). Quantitative PCR (qPCR) was performed with PowerUp SYBR Green master mix on 1 ng cDNA with a QuantStudio 6 Flex and analyzed with QuantStudio Real-Time PCR Software v1.3 (Applied Biosystems). Average Ct value from three technical replicates per sample was normalized to average Ct value of ribosomal protein L13a (*Rpl13a*) and *Gapdh* reference gene expression. -ΔΔCt values were determined in reference to average uninjured control or vehicle control ΔCt value. Validated primer sequences were obtained from PrimerBank where available (Wang et al., 2012). Additional primers were designed and validated for amplification efficiency by standard curve analysis. Single amplified products were noted from melting point analyses and agarose gel electrophoresis. Primer sequences are as follows: *Rpl13a* forward 5’-AGCCTACCAGAAAGTTTGCTTAC-3′, *Rpl31a* reverse 5’-GCTTCTTCTTCCGATAGTGCATC-3′, *Gapdh* forward 5’- AGGTCGGTGTGAACGGATTTG-3′, *Gapdh* reverse 5’- TGTAGACCATGTAGTTGAGGTCA-3′, *Gfap* forward 5′-GGGGCAAAAGCACCAAAGAAG-3′, *Gfap* reverse 5′-GGGACAACTTGTATTGTGAGCC-3′, *CD16* forward 5′-TGTTTGCTTTTGCAGACAGG-3′, *CD16* reverse 5′-GCACCGGTATTCTCCACTGT-3′, *CD32* forward 5′-AGAAGCTGCCAAAACTGAGG-3′, *CD32* reverse 5′-GTGGTTCTGGTAATCATGCTCTG-3′, *CD86* forward 5′-TGTTTCCGTGGAGACGCAAG-3′, *CD86* reverse 5′-TTGAGCCTTTGTAAATGGGCA-3′, *Arg1* forward 5′-CTCCAAGCCAAAGTCCTTAGAG-3′, *Arg1* reverse 5′-AGGAGCTGTCATTAGGGACATC-3′, *CD163* forward 5-ATGGGTGGACACAGAATGGTT-3′, *CD163* reverse 5′-CAGGAGCGTTAGTGACAGCAG-3′, *CD206* forward 5′-CTCTGTTCAGCTATTGGACGC-3′, *CD206* reverse 5′-CGGAATTTCTGGGATTCAGCTTC-3′, *Trem2* forward 5′-CTGGAACCGTCACCATCACTC-3′, *Trem2* reverse 5′-CGAAACTCGATGACTCCTCGG-3′.

### Analysis of cultured DRG neurons

Sciatic nerve crush was performed on adult *Nav1.8^Cre/+^; Tsc2^fl/+^; Rosa26-ZsGreen^GFP/+^* (control) and *Nav1.8^Cre/+^; Tsc2^fl/null^; Rosa26-ZsGreen^GFP/+^* (Tsc2 cKO) mice. After 3 d, mice were euthanized and transcardially perfused with Hanks’ balanced salt solution. Uninjured and injury-conditioned L4 DRG were isolated in Hanks’ balanced salt solution with 10 mM HEPES (HBSS-H). DRG were treated at 37°C with papain (15 U/ml, Worthington Biochemical) and collagenase (1.5 mg/ml, Sigma-Aldrich) in HBSS-H with intermittent HBSS-H washes. DRG were dissociated by trituration, passed through a 70-μm cell strainer, and 1/4 of resuspended cells were plated into two wells of a 24-well plate coated with 100 μg/ml poly-D-lysine and 3 μg/ml laminin. Cell were cultured in Neurobasal-A medium supplemented with B_27_ plus, glutaMAX, and penicillin/streptomycin (Thermo Fisher) at 37°C, 5% CO_2_.

After 24 h in culture, cells were fixed with 3% paraformaldehyde in PBS, washed with PBS, blocked with 5% donkey serum dissolved in PBSTx and stained with IB4 directly conjugated to Alexa Fluor 488 (1:250; Thermo Fisher Scientific catalog #I21411,) and Tubb3 antibody (1:1000; BioLegend catalog #802001, RRID:AB_291637). Cells were washed several times with PBS, incubated with fluorescent-conjugated secondary antibody (1:1000; Thermo Fisher Scientific) and DAPI (1:1000) diluted in blocking buffer, washed and mounted in ProLong Gold antifade mountant (Thermo Fisher Scientific). Images were taken with a Nikon TE-2000E compound microscope and Prior ProScan3 motorized stage, which automatically stitched images in Nikon Elements. Images were analyzed using FIJI software (NIH).

For percentage initiation, all neurons (Tubb3 and DAPI double-positive profiles) in a single well were counted for each biological replicate, categorized as IB4-positive, GFP-positive or IB4-negative, GFP-positive or GFP-negative, and the number of neurons initiating axon growth was determined. Axon initiation was defined as neurons with an axon greater than two cell diameters in length. Radial length was measured from the center of the neuron soma to the farthest point from the soma center that its axon crosses from both wells from uninjured DRG or from half of a well from injured DRG. Length measurements were only recorded from neurons that initiated axons as defined above.

### Behavioral analysis

Mechanical sensitivity was determined with calibrated von Frey filaments by using the up-and-down paradigm (Chaplan et al., 1994). Briefly, each mouse was placed individually under a polyacrylamide enclosure on a mesh base and allowed to acclimate for 3 h. The hind limbs were then tested with 0.32-g von Frey filaments. If the animal responded to the stimulus, the filament strength was decreased. Alternatively, if the animal did not respond, the filament strength was increased. The 50% withdrawal threshold was then calculated (Dixon, 1980). Testing was conducted preoperatively, and then semiweekly for the duration of the study beginning after the first postoperative week.

To assess cold allodynia, mice were placed individually under a polyacrylamide enclosure on a mesh base and allowed to acclimate for 3 h; 0.05–0.1 ml of acetone was applied to the plantar aspect of the left hindlimb. The animals were observed for 1 min. The total time of response was recorded. The right leg was then tested. Both hind limbs were tested for a total of five trials, and the total time was averaged. A positive response included licking of the plantar aspect of the leg or refusal to bear weight on the affected limb. Testing was conducted preoperatively, and then weekly for the duration of the study.

## Results

### Nociceptor deletion of Tsc2 improves axon regeneration within 2 d of nerve injury in an mTORC1-dependent manner

**Figure 1.**
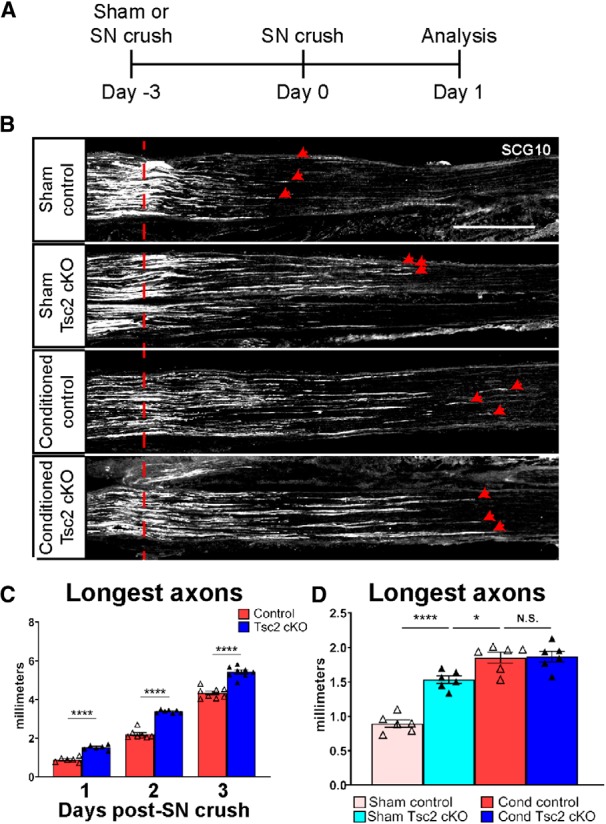
Neuronal deletion of Tsc2 improves axon regeneration within the first 2 d following nerve injury. ***A***, Scheme of *in vivo* conditioning experiment. SN denotes sciatic nerve. ***B***, SCG10 immunostaining of sciatic nerves 1 d after crush injury in control and Tsc2 cKO mice with (conditioned) or without (sham) a conditioning injury. Red dotted line denotes the crush site while red arrowheads point to the three longest axons. Scale bar: 500 μm. ***C***, Quantification of average of the ten longest axons at 1, 2, and 3 d following a single sciatic nerve crush. ***D***, Quantification of average of the ten longest axons at 1 d after sciatic nerve crush with (cond) and without (sham) a conditioning injury 3 d prior from B. N.S., not significant, *p < 0.05, ****p < 0.0001. Extended Data Figure 1-1 shows data values of mean and SEM, number of replicates, statistical tests, and values for all comparisons.

Long-distance axon regeneration requires sustained protein translation, and mTORC1 is a potent regulator of cellular growth and protein translation (Bradke et al., 2012; Saxton and Sabatini, 2017; Terenzio et al., 2018). Therefore, we hypothesized that activating mTORC1 in nociceptive neurons, which do not regenerate as well as non-nociceptive neurons (Kalous and Keast, 2010), could both establish and maintain enhanced axon growth after a peripheral nerve injury. We analyzed axon regeneration in mice in which Tsc2 deletion was mediated by Nav1.8-Cre (also known as SNS-Cre), which is predominantly expressed in nociceptors. Nav1.8-Cre drives specific, perinatal expression of Cre recombinase in ∼75% of all DRG neurons, including >90% of C-fiber neurons and ∼40% of A-fiber neurons, with no expression in non-neuronal cells or CNS neurons (Agarwal et al., 2004; Shields et al., 2012). Mice with perinatal Tsc2 deletion as a result of Nav1.8-Cre are denoted as Tsc2 cKO. Using SCG10 to specifically label regenerating axons (Shin et al., 2014), we found that Tsc2 cKO mice showed improved axon regeneration 1 d after sciatic nerve crush, with further improvement by 2 d (sham/1 d: ctrl 0.89 ± 0.05 mm vs Tsc2 cKO 1.53 ± 0.05 mm; 2 d: ctrl 2.21 ± 0.09 mm vs Tsc2 cKO 3.40 ± 0.03 mm; Fig. 1*B*,*C*), consistent with our previous results (Abe et al., 2010). However, analysis of axon regeneration 3 d after nerve crush showed no additional improvement (3 d: ctrl 4.42 ± 0.11 mm vs Tsc2 cKO 5.58 ± 0.09 mm; Fig. 1*C*). These data suggest that Tsc2 deletion may only have an early effect on axon regeneration.

10.1523/ENEURO.0168-19.2019.f1-1 Extended Data Figure 1-1 Data values, biological replicates, and statistics supporting Figure 1. Download Figure 1-1, XLSX file.

After a peripheral nerve injury, retrograde signals from the injury site induce a transcription-dependent regeneration program in DRG neurons within 24–48 h, whereby the regenerative growth rate of axons increases with the changes in neuronal gene expression (Smith and Skene, 1997; Mahar and Cavalli, 2018). We thus tested whether the 1-d axon growth rate 3 d after a conditioning injury is similar in control and Tsc2 cKO mice (Fig. 1*A*). In control mice, injury-conditioned axons grew significantly longer than sham, as expected (Shin et al., 2012). However, injury-conditioned axon growth was not further improved by deletion of Tsc2 (Fig. 1*B*,*D*). These data suggest that the axon elongation rate of control axons catches up to the initially enhanced rate of Tsc2-deficient neurons by 3 d post-injury.

**Figure 2.**
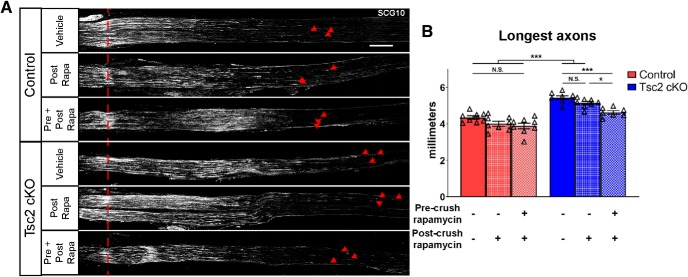
mTORC1 inhibition before injury is required to suppress the enhanced axon regeneration in Tsc2 cKO mice. ***A***, Injured sciatic nerves stained with SCG10 to label regenerating axons 3 d after crush. Mice were treated daily with vehicle or rapamycin beginning 3 d before injury. Vehicle and Pre + Post Rapa denotes vehicle and rapamycin treatment, respectively, for all 6 d. Post Rapa denotes vehicle treatment before injury and rapamycin treatment post-injury. Red dotted line denotes the crush site while red arrowheads point to the three longest axons. Scale bar: 500 μm. ***B***, Quantification of average of the ten longest axons 3 d following sciatic nerve crush from A. N.S., not significant, *p < 0.05, ***p < 0.001. Extended Data Figure 2-1 shows data values of mean and SEM, number of replicates, statistical tests, and values for all comparisons.

As Tsc2 deletion in nociceptors increases mTORC1 activity (Carlin et al., 2018), we sought to confirm that enhanced axon growth in Tsc2-deficient neurons resulted from increased mTORC1 signaling. To test this, we compared axon regeneration 3 d after a sciatic nerve crush in control and Tsc2 cKO mice that were treated daily with vehicle or rapamycin starting at the time of injury. Consistent with a previous study (Chen et al., 2016), we found that axon regeneration was insensitive to rapamycin in control mice (Fig. 2). Surprisingly, we also observed that the enhanced regeneration in Tsc2 cKO mice was also insensitive to rapamycin treatment post-injury (Fig. 2), suggesting that acute mTORC1 signaling does not have a significant role in improving regeneration. As Tsc2 deletion is perinatal while injury occurs in adult mice, we hypothesized that chronic mTORC1 activation may induce a pro-regenerative state in neurons before injury. To test this hypothesis, we assessed axon regeneration 3 d after a sciatic nerve crush in control and Tsc2 cKO mice that were treated daily with vehicle or rapamycin starting 3 d before injury (before and after crush). Rapamycin treatment starting 3 d before injury in Tsc2 cKO mice showed a significant reduction of axon growth to near vehicle-treated control lengths (Fig. 2). Axon growth in control mice with pre-crush and post-crush rapamycin treatment was slightly reduced compared to similarly treated Tsc2 cKO mice (*p* = 0.0009), which is consistent with incomplete inhibition mTORC1 signaling by rapamycin. Taken together, these results suggest that nociceptor deletion of Tsc2 induces an mTORC1-dependent, pro-regenerative program uninjured neurons.

10.1523/ENEURO.0168-19.2019.f2-1 Extended Data Figure 2-1 Data values, biological replicates, and statistics supporting Figure 2. Download Figure 2-1, XLSX file.

### Nociceptor deletion of Tsc2 induces pro-regenerative gene expression in uninjured DRG neurons

**Figure 3.**
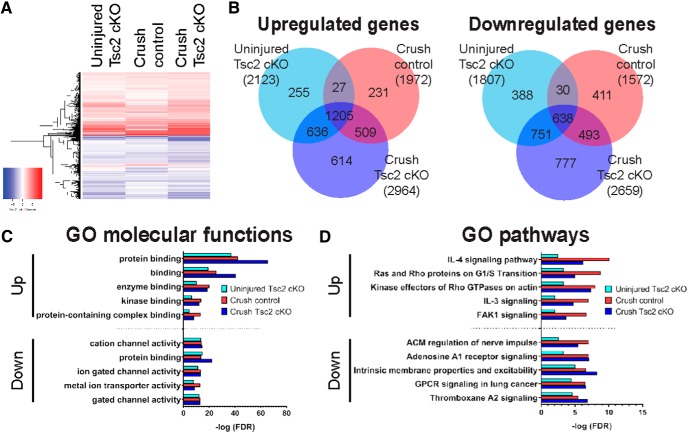
Similar molecular pathways are induced in Nav1.8-positive neurons by injury and by Tsc2 deletion. ***A***, Heat map of differentially expressed genes in FACS-sorted, Nav1.8-positive neurons for each condition relative to uninjured control (*n* = 4 total; *n* = 2 male; *n =* 2 female; 8600 genes with adjusted *p* < 0.05 in at least one condition). ***B***, Venn diagrams showing differentially expressed genes (adjusted *p* < 0.05, log2 fold change >0.5 or <–0.5) relative to uninjured control neurons for each condition. Total number of differentially expressed genes in each condition in parenthesis. ***C***, ***D***, Top five GO molecular functions and pathways categories differentially regulated in crush control neurons and Tsc2 cKO. Extended Data Figure 3-1 shows genes differentially expressed (adjusted *p* < 0.05, log2 fold change >0.5 or <–0.5) under at least one condition relative to uninjured control neurons.

As our regeneration studies suggest that Tsc2-deficient neurons are in a pro-regenerative state, we compared the gene expression profiles of uninjured and injured control and Tsc2 cKO neurons. We crossed control and Tsc2 cKO mice with a *Rosa26-ZsGreen* reporter line to label all Nav1.8-positive neurons (Madisen et al., 2010; Carlin et al., 2018). Uninjured and injured L4 DRG were dissociated 3 d after sciatic nerve injury, and three technical replicates of 100 GFP-positive neurons were sorted by flow cytometry for library preparation and RNA-seq. This method enriched for neurons, specifically nociceptors, and previous differential expression analysis comparing uninjured control and Tsc2 cKO neurons uncovered dysregulation of a large number of sensory behavior-related genes and ion channels (Carlin et al., 2018). Here, we identified 8600 genes as differentially expressed by Tsc2 deletion and/or injury compared to uninjured control, with similar gene expression changes in each condition (adjusted *p* < 0.05; Fig. 3*A*; Extended Data Fig. 3-1). Of those genes, ∼7000 exhibited a log2 fold change >0.5 or <–0.5, and were used for further downstream analysis. Specifically, 62.5% and 42.5% of genes that were upregulated and downregulated after injury, respectively, exhibited similar dysregulation in uninjured Tsc2 cKO neurons (Fig. 3*B*). Additionally, we compared differentially regulated GO molecular functions and pathways using thresholded differentially expressed genes under each condition relative to uninjured control neurons. The top five differentially regulated GO molecular functions and pathways after injury in control neurons were similarly dysregulated in uninjured Tsc2 cKO neurons, albeit to a lesser extent in several cases (Fig. 3*C*,*D*). While there are clear similarities between Tsc2 deletion and nerve injury, these analyses show that the overlap is not complete, suggesting that Tsc2 deletion may induce a partial pro-regenerative program.

10.1523/ENEURO.0168-19.2019.f3-1 Extended Data Figure 3-1 Genes with differential expression relative to uninjured control. Download Figure 3-1, XLSX file.

**Figure 4.**
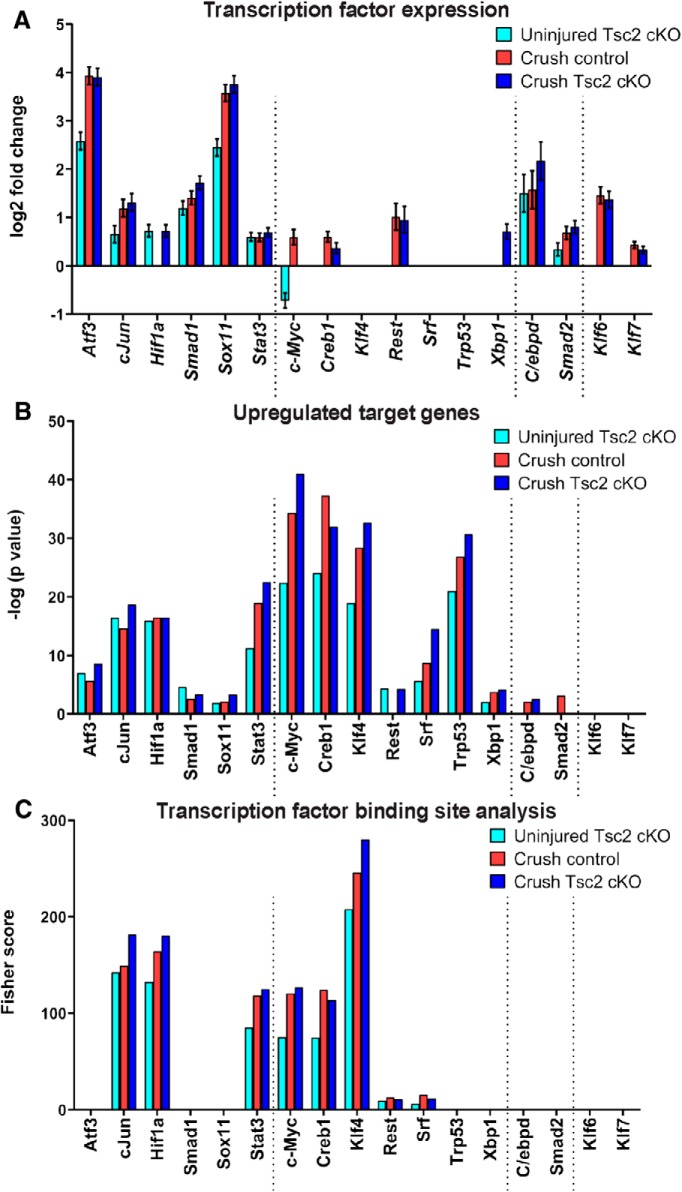
Tsc2 deletion upregulates expression of RATFs and their target genes in the absence of injury. ***A***, Expression levels of RATFs in FACS-sorted Nav1.8-positive neurons. Differential expression is noted relative to uninjured control. Differential expression with adjusted *p* > 0.05 is reported as 0 values. ***B***, Interactome analysis of upregulated genes from FACS-sorted, Nav1.8-positive neurons assessed for preferential upregulation of RATF target genes identified by Metacore software (threshold FDR < 0.05). Interactions with *p* > 0.05 are reported as 0 values. ***C***, Transcription factor binding site analysis using oPOSSUM 3.0 of upregulated genes relative to uninjured control in FACS-sorted Nav1.8-positive neurons. Dotted lines denote classes of RATFs defined in Results. Log2 fold change and *p* values for transcription factor expression changes can be found in Extended Data Figure 3-1.

A number of regeneration-associated transcription factors (RATFs) have been identified and characterized for necessity and/or sufficiency to promote axon regeneration in the PNS (Patodia and Raivich, 2012; Mahar and Cavalli, 2018; Oh et al., 2018). Using these gene lists, we assessed whether Tsc2 deletion affected gene expression of RATFs in a similar manner as nerve injury in FACS-sorted, Nav1.8-positive neurons. Indeed, many RATFs exhibited increased expression after nerve injury in control neurons, including *Atf3*, *cJun*, *Smad1*, *Sox11*, *Stat3*, *Creb1*, *Rest*, *C/ebpd*, *Smad2*, *Klf6*, and *Klf7*. Tsc2 deletion was sufficient to increase expression of those RATFs in the absence of injury with the exceptions of *Creb1*, *Rest*, *Klf6*, and *Klf7* (Fig. 4*A*). Our RNA-seq analysis uncovered significant similarities of RATF expression changes by Tsc2 deletion or nerve injury.

To assess whether RATF activity was also affected by Tsc2 deletion and/or nerve injury, we assessed the upregulated genes under each condition relative to uninjured control for overrepresentation of RATF target genes. Target genes were identified from Metacore software databases (Clarivate Analytics). We observed overrepresentation of RATF target genes for most RATFs after both injury and Tsc2 deletion, including for RATFs that exhibited no change of their own expression (Fig. 4*A*,*B*). Similarly, transcription factor binding site analysis of upregulated genes compared to uninjured control uncovered similar RATF binding profiles after nerve injury or Tsc2 deletion (Fig. 4*C*). From these analyses, we noted four classes of RATFs in relation to their activity and expression in uninjured Tsc2 cKO neurons. In the first class (Fig. 4, far left), upregulated expression of the RATF and overrepresentation of its target genes were observed in Tsc2 cKO neurons. This group includes Atf3, cJun, Hif1a, Smad1, Sox11, and Stat3. Notably, expression of many RATFs in this category was upregulated to a lesser extent as a result of Tsc2 deletion than after nerve injury. The second class of RATFs exhibited increased activity noted from overrepresentation of upregulated RATF target genes (Fig. 4, middle left), however RATF expression itself was unaffected or even reduced in uninjured Tsc2 cKO neurons. This group includes c-Myc, Creb1, Klf4, Rest, Srf, Trp53, and Xbp1. Rest is an intriguing member of this list as it is a transcriptional repressor and only shows upregulated expression of target genes as a result of Tsc2 deletion but not after injury. Similar to relative expression of RATFs in the first class, overrepresentation of upregulated target genes in this second class was more extensive after nerve injury than as a result of Tsc2 deletion. The third class of RATF contains Smad2 and C/ebpd (Fig. 4, middle right). No overrepresentation of these RATF target genes was observed as a result of Tsc2 deletion despite an increase in their own expression. This class shows activity in Tsc2 cKO neurons that is contradictory to their activity post-injury in control neurons. Finally, Klf6 and Klf7 expression was increased after injury but not Tsc2 deletion while their overrepresentation of their upregulated target genes was not notably affected under any condition (Fig. 4, far right). This may be a result of incomplete profiling of their target genes in the Metacore software database. Together, these data show that expression and activity of several known RATFs are similarly affected by nociceptor deletion of Tsc2 and by nerve injury, suggesting that Tsc2 deletion primes neurons for axon regeneration even in the absence of an injury.

Our data show that the rate of axon growth 3 d following nerve injury was unaffected by Tsc2 deletion (Fig. 1*B–D*). As such, we predicted that expression profiling of RATFs and their upregulated target genes in injured DRG neurons would not show additive dysregulation as a result of Tsc2 deletion. Indeed, for virtually all RATFs analyzed, RATF and target gene expression were affected to similar degrees by injury in both control and Tsc2 cKO neurons (Fig. 4*A–C*). Therefore, we conclude that Tsc2 deletion in uninjured DRG neurons establishes a partial pro-regenerative gene expression landscape, but Tsc2 deletion does not further enhance the regenerative state of injured neurons.

### Nociceptor deletion of Tsc2 upregulates cJun and Atf3 expression in IB4-positive neurons

**Figure 5.**
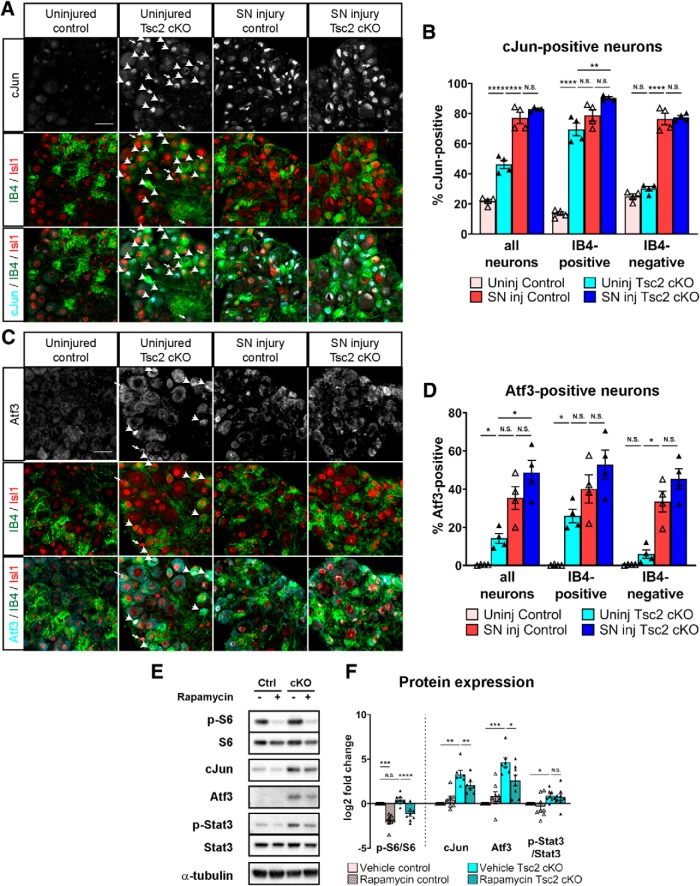
Nociceptor deletion of Tsc2 preferentially upregulates cJun and Atf3 expression in IB4-positive neurons. ***A***, Immunohistochemistry of L4 DRG contralateral and ipsilateral to a sciatic nerve transection (SN injury) at 3 d post-injury stained for cJun, Isl1 (all neurons), and IB4. Arrows point to cJun-positive, IB4-negative neurons, and arrowheads point to cJun, IB4 double-positive neurons in uninjured Tsc2 cKO DRG. Scale bars: 50 μm. ***B***, Quantification of percentage of cJun-positive neurons from ***A***. ***C***, Immunohistochemistry of L4 DRG for Atf3, Isl1 (all neurons) and IB4. Arrows point to Atf3-positive, IB4-negative neurons and arrowheads point to Atf3, IB4 double-positive neurons in uninjured Tsc2 cKO DRG. Scale bars: 50 μm. ***D***, Quantification of percentage of Atf3-positive neurons from ***C***. ***E***, Western blotting of uninjured control and Tsc2 cKO L4/L5 DRG from mice receiving daily vehicle or rapamycin treatment for 3 d. ***F***, Quantification of protein expression from ***E***. Log2 fold change relative to uninjured control from the same biological replicate. N.S., not significant, *p < 0.05, **p < 0.01, ***p < 0.001, ****p < 0.0001. Extended Data Figure 5-1 shows data values of mean and SEM, number of replicates, statistical tests, and values for all comparisons.

cJun and Atf3 have been shown to be both necessary and sufficient to promote axon regeneration in peripheral nerves (Raivich et al., 2004; Seijffers et al., 2007; Ruff et al., 2012; Fagoe et al., 2015; Chandran et al., 2016; Gey et al., 2016), and mTORC1 signaling affects expression of these RATFs in other cell types (Nie et al., 2015; Norrmén et al., 2018). To validate our sequencing results, we counted cJun-positive and Atf3-positive neuronal nuclei in uninjured and injured DRG 3 d after a sciatic nerve transection. We observed an increased number of cJun-positive and Atf3-positive neuronal nuclei in uninjured Tsc2 cKO DRG, but notably less than the amount of positive nuclei after an injury (Fig. 5*A–D*), consistent with our RNA-seq analysis (Fig. 4*A*). These data suggest a cell type-specific response to Tsc2 deletion. Calcitonin gene-related peptide (CGRP) and *G. simplicifolia* IB4 are common markers to positively identify peptidergic and nonpeptidergic classes of nociceptors, respectively. Tsc2 deletion alters the distribution of these neuronal subtypes with an expansion in the number of IB4-positive neurons and reduction in the number of CGRP-positive neurons as well as reduced CGRP expression (Carlin et al., 2018). To positively identify nonpeptidergic nociceptors and determine whether cJun or Atf3 expression was preferentially upregulated in these neurons, we labeled DRG for IB4 binding. After injury, cJun and Atf3 nuclear localization increased in both IB4-positive and IB4-negative neurons to similar extents; however, in uninjured Tsc2 cKO DRG the vast majority of neurons showing increased cJun and Atf3 nuclear localization were IB4-positive (Fig. 5*A–D*). Despite the ubiquitous expression of Tsc2 in DRG neurons, IB4-positive neurons preferentially upregulated cJun and Atf3 expression in response to Tsc2 deletion.

10.1523/ENEURO.0168-19.2019.f5-1 Extended Data Figure 5-1 Data values, biological replicates, and statistics supporting Figure 5. Download Figure 5-1, XLSX file.

As rapamycin pre-treatment reduced the enhanced axon regeneration in Tsc2 cKO mice, we assessed whether increased cJun or Atf3 expression was affected by 3 d of daily rapamycin treatment in uninjured control and Tsc2 cKO DRG. Phosphorylation of ribosomal protein S6 is a downstream marker of mTORC1 activity, and it was strongly reduced by rapamycin treatment in both control and Tsc2 cKO DRG (Fig. 5*E*,*F*). Rapamycin treatment also partially reduced protein expression of cJun and Atf3 in Tsc2 cKO DRG (Fig. 5*E*,*F*), suggesting that the pro-regenerative gene expression induced by Tsc2 deletion can be suppressed by mTORC1 inhibition.

### Nociceptor deletion of Tsc2 induces pro-regenerative non-neuronal responses

**Figure 6.**
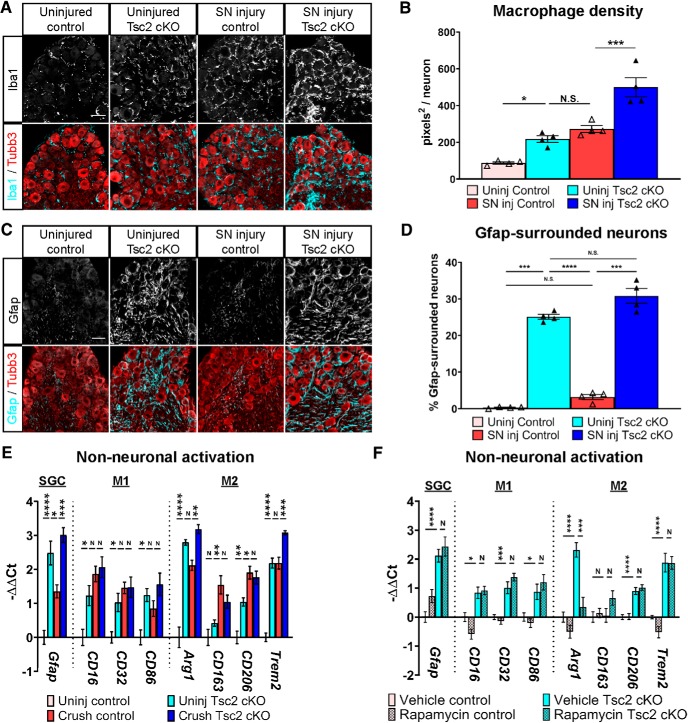
Nociceptor deletion of Tsc2 activates macrophages and satellite glial cells in DRG. ***A***, Immunohistochemistry of L4 DRG contralateral and ipsilateral to a sciatic nerve transection (SN injury) at 3 d post-injury stained for Iba1 and Tubb3. Scale bar: 50 μm. ***B***, Quantification of macrophage density by Iba1-positive area per neuron from ***A***. ***C***, Immunohistochemistry of L4 DRG contralateral and ipsilateral to a sciatic nerve transection (SN injury) at 3 d post-injury stained for Gfap and Tubb3. Scale bar: 50 μm. ***D***, Quantification of percentage of neurons surrounded by Gfap staining on at least two sides from ***C***. ***E***, qPCR of L4/L5 DRG contralateral or ipsilateral to sciatic nerve crush at 3 d post-injury for markers of activated satellite glial cells (SGC), M1, or M2 macrophages. ***F***, qPCR for markers of macrophage or SGC activation in L3 DRG from mice receiving daily vehicle or rapamycin treatment for 3 d. Normalized to uninjured control. N.S. or N, not significant, *p < 0.05, **p < 0.01, ***p < 0.001, ****p < 0.0001. Extended Data Figure 6-1 shows data values of mean and SEM, number of replicates, statistical tests, and values for all comparisons.

Recent studies have revealed a role for non-neuronal cells in promoting axon regeneration, especially macrophages. Macrophages infiltrate and become activated in DRG after nerve injury, and this process is necessary and sufficient to promote axon outgrowth similar to injury conditioning (Kwon et al., 2013, 2015; Niemi et al., 2013, 2016; Krishnan et al., 2018; Zigmond and Echevarria, 2019). We thus assessed the density of Iba1-positive macrophages in the DRG after injury or as a result of neuronal Tsc2 deletion. Tsc2 deletion and injury both increased macrophage density to similar extents, and this phenotype was additive in the injured Tsc2 cKO DRG (Fig. 6*A*,*B*). It has been previously reported that the predominant activation state of DRG macrophages after injury is the M2 phenotype (Kwon et al., 2015; Niemi et al., 2016). To determine the phenotype of DRG macrophages as a result of neuronal Tsc2 deletion, we analyzed M1 and M2 macrophage markers by qPCR of whole DRG. The expression levels of M1 markers *CD16*, *CD32*, and *CD86* as well as the M2 markers *Arg1*, *CD206*, and *Trem2* were upregulated by both Tsc2 deletion and injury, contrary to a previous study showing an M2 bias at the same time point (Fig. 6*E*; Kwon et al., 2013). The M2 marker *CD163* was upregulated only after injury but not by Tsc2 deletion suggesting that the M2 phenotype may be incomplete. Additional markers such as *Nos2* and *Il10* were undetectable in uninjured control DRG. Consistent with the increased macrophage density in injured Tsc2 cKO DRG, we also observed further upregulation of *Arg1* and *Trem2* expression in injured Tsc2 cKO DRG compared to injured control DRG (Fig. 6*E*).

10.1523/ENEURO.0168-19.2019.f6-1 Extended Data Figure 6-1 Data values, biological replicates, and statistics supporting Figure 6. Download Figure 6-1, XLSX file.

Ccr2-mediated activation of M2 macrophages via Ccl2 signaling is both necessary and sufficient to promote axon regeneration (Kwon et al., 2015; Niemi et al., 2016). To determine whether Ccr2 activation is responsible for macrophage recruitment and activation in uninjured Tsc2 cKO DRG, we assessed the differential gene expression of Ccr2 ligands *Ccl2*, *Ccl7*, *Ccl8*, and *Ccl11* in our RNA-seq dataset (Extended Data Fig. 3-1). *Ccl2*, *Ccl7*, and *Ccl11* were not differentially expressed after injury, and *Ccl2* expression was reduced while *Ccl7* and *Ccl11* expression remained unchanged in uninjured Tsc2 cKO neurons compared to uninjured control neurons (*Ccl2*: –2.1 ± 0.6 log2 fold change in uninjured Tsc2 cKO, adjusted *p* = 0.0037; *Ccl2*: –0.8 ± 0.6 log2 fold change in crush control, adjusted *p* = 0.3456). However, *Ccl8* expression was increased under all conditions compared to uninjured control neurons (*Ccl8*: 2.2 ± 0.8 log2 fold change in uninjured Tsc2 cKO, adjusted *p* = 0.0190; *Ccl8*: 3.5 ± 0.8 log2 fold change in crush control, adjusted *p* = 9.18E-05), suggesting that *Ccl8* may have a role in macrophage recruitment and activation in DRG as a result of nerve injury and/or neuronal Tsc2 deletion. Together, these data show that in the absence of an injury, neuronal deletion of Tsc2 induces a non-neuronal response in DRG with macrophage recruitment and activation similar to an injured state.

Although the exact role of satellite glial cells in axon regeneration remains unclear, these cells encircle DRG neuron cell bodies and react to nerve injury by upregulating Gfap expression (Fenzi et al., 2001; Hanani, 2005; Xie et al., 2009; Ji et al., 2013; Christie et al., 2015; Krishnan et al., 2018). We observed a dramatic increase in Gfap expression surrounding neurons in response to Tsc2 deletion, well beyond its expression 3 d post-injury in control DRG, by both qPCR and immunohistochemistry (Fig. 6*C–E*). These results suggest that satellite glial cells are strongly activated by neuronal Tsc2 deletion. In addition to the region surrounding the neuronal cell bodies, we also observed increased expression in regions enriched for axons, suggesting increased Gfap expression in Schwann cells as well.

To assess whether non-neuronal activation may contribute to the enhanced regeneration in Tsc2 cKO mice, we assessed if these phenotypes were sensitive to mTORC1 inhibition by rapamycin in uninjured L3 DRG. Vehicle-treated Tsc2 cKO L3 DRG showed similar upregulation of *Gfap*, M1 and M2 markers as untreated L4 DRG (Fig. 6*E*,*F*). Surprisingly, *Gfap* expression as well as M1 macrophage activation in Tsc2 cKO DRG were unaffected by rapamycin treatment (Fig. 6*F*). M2 macrophage markers *CD206* and *Trem2* in Tsc2 cKO DRG were unaffected by rapamycin treatment, however *Arg1* expression returned to vehicle-treated control levels (Fig. 6*F*). This suggests a complex effect of mTORC1 inhibition on the M2 macrophage phenotype. Stat3 was shown to promote enhanced axon growth downstream of Ccr2-mediated macrophage activation in DRG (Niemi et al., 2016). Consistent with maintained macrophage activation in rapamycin-treated Tsc2 cKO DRG, we did not observe a reduction in Stat3 phosphorylation compared to vehicle-treated Tsc2 cKO DRG (Fig. 5*E*,*F*). Together, these data suggest that mTORC1 inhibition in Tsc2 cKO mice incompletely suppresses the upregulated neuronal and non-neuronal pro-regenerative gene expression.

### Nociceptor deletion of Tsc2 increases axon length but not the percentage of neurons initiating axons *in vitro*

**Figure 7.**
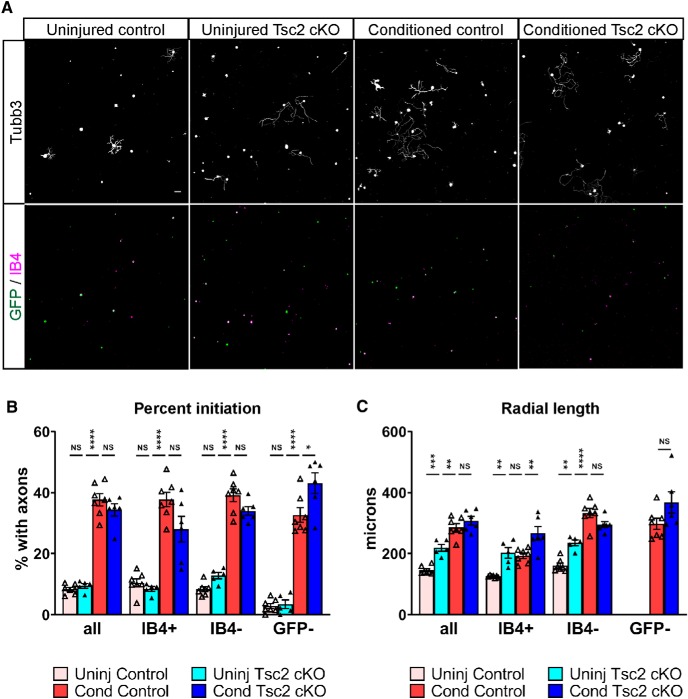
Nociceptor deletion of Tsc2 increases axon length but not percentage initiation in cultured neurons. ***A***, Uninjured and injury-conditioned (3 d) L4 DRG neurons from control and Tsc2 cKO mice crossed with *Rosa26-ZsGreen^GFP/+^* cultured for 24 h and stained for neuronal tubulin (Tubb3) and IB4. Scale bar: 50 μm. ***B***, ***C***, Quantification of the percentage of cultured neurons that grow axons (***B***) and the radial length of those axons (***C***) from ***A***. Radial length of uninjured GFP– neurons is not reported due to the number of neurons growing axons being too low to be representative. N.S., not significant, *p < 0.05, **p < 0.01, ***p < 0.001, ****p < 0.0001. Extended Data Figure 7-1 shows data values of mean and SEM, number of replicates, statistical tests, and values for all comparisons.

As neuronal Tsc2 deletion and nerve injury activate similar neurons and non-neuronal responses, we tested whether these conditions have similar effects on axon growth in culture. A conditioning injury increases both the percentage of neurons that initiate axons as well as the length of those axons (Smith and Skene, 1997; Lankford et al., 1998). We therefore dissociated L4 DRG neurons from control and Tsc2 cKO adult mice with or without a conditioning injury and analyzed axon growth after 24 h *in vitro*. Analysis of all neurons from uninjured Tsc2 cKO DRG showed no change in the percentage of neurons initiating axons despite increased axon length, while a conditioning injury increased both parameters relative to uninjured control neurons (Fig. 7). These data suggest that the pro-regenerative program induced by Tsc2 deletion is incomplete.

10.1523/ENEURO.0168-19.2019.f7-1 Extended Data Figure 7-1 Data values, biological replicates, and statistics supporting Figure 6. Download Figure 7-1, XLSX file.

As Nav1.8-Cre is expressed predominantly, but not exclusively, in nociceptors and these neurons have been shown to be insensitive to injury conditioning (Agarwal et al., 2004; Kalous and Keast, 2010), we sought to confirm that nociceptors exhibit improved axon growth as a result of Tsc2 deletion. We used control and Tsc2 cKO mice crossed with *Rosa26-ZsGreen^GFP/+^* to analyze axon growth in culture in GFP-positive neurons that are either IB4-positive or IB4-negative, as well as in GFP-negative neurons. Both IB4-positive and IB4-negative axons grew longer as a result of Tsc2 deletion, however neither population showed an increased percentage of neurons initiating axons (Fig. 7). Surprisingly, Tsc2 deletion and conditioning injury produced an additive phenotype in IB4-positive axon length (Fig. 7*C*). Contrary to previous literature (Kalous and Keast, 2010), injury conditioning increased both the percentage of initiating axons as well as length in IB4-positive as well as IB4-negative control neurons (Fig. 7). While multiple sensory neuron subtypes including IB4-positive nociceptors showed improved axon growth as a result of Tsc2 deletion, the induced pro-regenerative program in insufficient to fully phenocopy injury conditioning.

The pro-regenerative activation of non-neuronal cells as a result of nociceptor deletion of Tsc2 (Fig. 6) may induce a conditioning effect on Nav1.8-negative neurons. To assess this possibility, we analyzed *in vitro* axon growth in GFP-negative neurons from Tsc2 cKO mice where Cre expression, and consequently Tsc2 deletion, was not induced. This GFP-negative population did not exhibit an increased percentage of neurons with axons from uninjured Tsc2 cKO mice relative to control mice (Fig. 7*A*,*B*). Length measurement of GFP-negative neurons from uninjured DRG could not be obtained due to the very low numbers of these cells initiating axons. However, injury conditioning improved both percentage of initiating neurons as well as axon length in GFP-negative neurons (Fig. 7), suggesting that Tsc2 deletion does not induce a conditioning effect on wild type neurons in the same DRG.

### Tsc2 deletion in adult mice induces a pro-regenerative DRG landscape and initial regenerative axon growth without improving functional recovery

**Figure 8.**
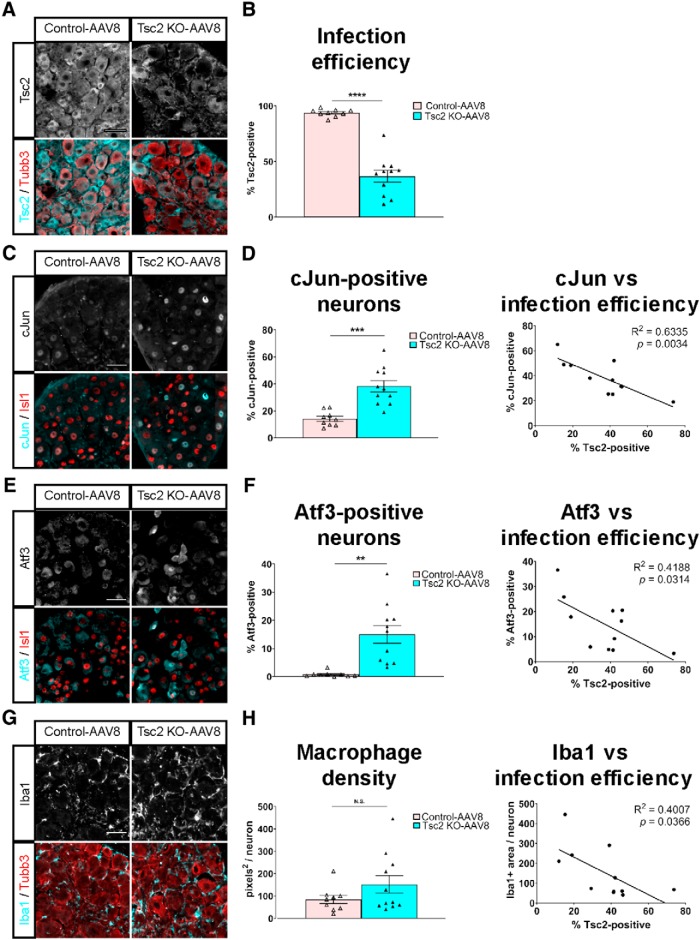
AAV8-mediated deletion of Tsc2 in adult mice induces a pro-regenerative environment in DRG. ***A***, Immunohistochemistry of uninjured L4 DRG stained for Tsc2 and Tubb3. Mice had intrathecal injections three weeks before a sciatic nerve crush. Uninjured DRG were isolated 3 d post-injury. ***B***, Quantification of percentage of Tsc2-positive neurons from ***A***. ***C***, Immunohistochemistry of uninjured L4 DRG stained for cJun and Isl1. ***D***, Quantification of percentage of cJun-positive neurons from ***C*** as well as linear regression of cJun expression and infection efficiency for Tsc2 KO-AAV8 mice. ***E***, Immunohistochemistry of uninjured L4 DRG stained for Atf3 and Isl1. ***F***, Quantification of percentage of Atf3-positive neurons from ***E*** as well as linear regression of Atf3 expression and infection efficiency for Tsc2 KO-AAV8 mice. ***G***, Immunohistochemistry of uninjured L4 DRG stained for Iba1 and Tubb3. Image from DRG with high infection efficiency. ***H***, Quantification of macrophage density by Iba1-positive area per neuron from ***G*** as well as linear regression of macrophage density and infection efficiency for Tsc2 KO-AAV8 mice. N.S., not significant, **p < 0.01, ***p < 0.001, ****p < 0.0001. Scale bars: 50 μm. Extended Data Figure 8-1 shows data values of mean and SEM, number of replicates, statistical tests, and values for all comparisons.

Nociceptor-specific deletion of Tsc2 occurred perinatally in Tsc2 cKO mice and was accompanied by significant gene expression changes in adult DRG neurons (Figs. 3, 4; Agarwal et al., 2004; Carlin et al., 2018). To determine whether a pro-regenerative DRG environment could be induced by adult deletion of Tsc2, both control (*Tsc2^fl/+^*) and experimental (*Tsc2^fl/fl^*) adult mice were injected intrathecally with an adeno-associated virus (AAV8) expressing Cre recombinase, hereafter referred to as control-AAV8 and Tsc2 KO-AAV8, respectively. AAVs have limited cell-type specificity, and Cre-mediated GFP reporter expression from the *Rosa26* locus confirmed that neurons as well as non-neuronal cells were infected in DRG. In control mice, Tsc2 protein expression was nearly ubiquitous in DRG neurons. In contrast, we observed a substantial reduction in the percentage of Tsc2-expressing neurons 3.5 weeks post-infection in Tsc2 KO-AAV8 mice, with > 50% deletion in most cases (Fig. 8*A*,*B*). To confirm that gene deletion occurred in nociceptive neurons as a result of AAV8-Cre infection, we quantified the percentage of IB4-positive and CGRP-positive neurons that co-express Cre-mediated GFP reporter expression. In Tsc2 KO-AAV8 mice, we observed 61.0 ± 5.9% of all L4 DRG neurons expressed GFP as a result of AAV8-Cre infection. Similarly, 64.6 ± 7.6% of IB4-positive neurons and 64.9 ± 6.6% of CGRP-positive neurons co-express GFP (all vs IB4: *p* = 0.1919; all vs CGRP: *p* = 0.2208; paired *t* test; *N* = 5), suggesting that AAV8-Cre can infect nociceptive neurons as efficiently as non-nociceptive neurons in DRG. Although we cannot exclude the possibility that Tsc2 deletion also occurred in non-neuronal cells, intrathecal injections of AAV8-Cre efficiently deleted Tsc2 in lumbar DRG neurons of adult mice.

10.1523/ENEURO.0168-19.2019.f8-1 Extended Data Figure 8-1 Data values, biological replicates, and statistics supporting Figure 8. Download Figure 8-1, XLSX file.

To test whether Tsc2 deletion in adult mice induces a pro-regenerative landscape in the DRG, we assessed cJun and Atf3 expression as well as macrophage density in uninjured DRG of control-AAV8 and Tsc2 KO-AAV8 mice 3.5 weeks post-infection. Adult deletion of Tsc2 induced an upregulation of cJun-positive and Atf3-positive nuclei with a correlation between the amount of RATF-positive neurons and infection efficiency (Fig. 8*C–F*). In contrast, increased macrophage density was only noted in mice with the highest levels of Tsc2 deletion (Fig. 8*G*,*H*; images from mice with high infection efficiency). Macrophage density appeared similar in control-AAV8 and Tsc2 KO-AAV8 with > 40% Tsc2-positive neurons, suggesting a threshold level of gene deletion is required for non-neuronal activation. These results show that deletion of Tsc2 in adult mice is sufficient to induce a pro-regenerative environment in DRG, albeit less potently than genetic deletion in Tsc2 cKO mice.

**Figure 9.**
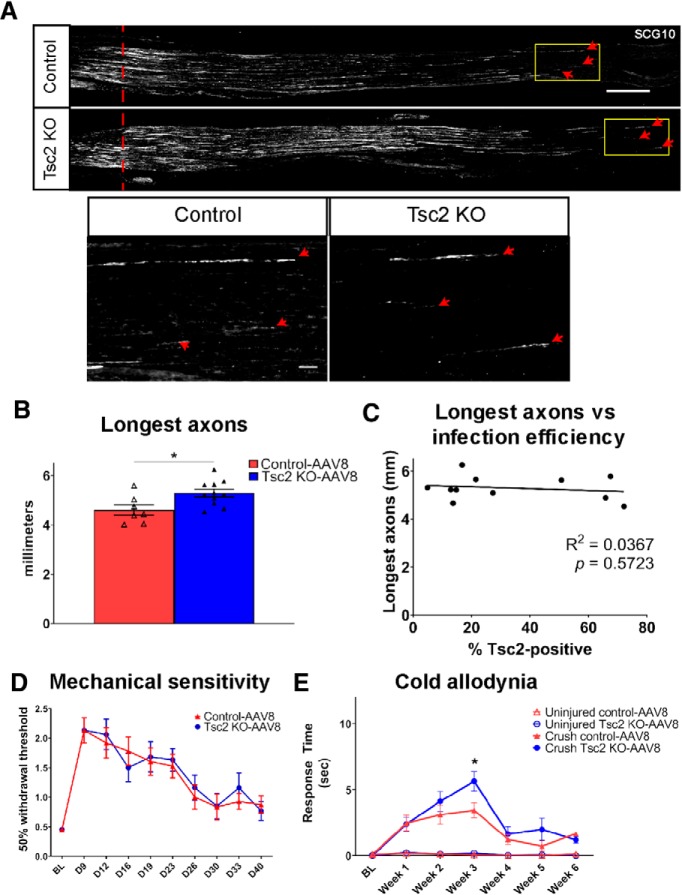
AAV8-mediated deletion of Tsc2 improves initial axon regeneration but not functional recovery after sciatic nerve crush injury. ***A***, Injured sciatic nerve stained with SCG10 to label regenerating axons 3 d after crush. Larger images of yellow boxed region shown below whole nerve images. Red dotted line denotes the crush site while red arrowheads point to the three longest axons. Scale bars: 500 μm (top) and 50 μm (bottom). ***B***, ***C***, Quantification of average of the ten longest axons 3 d following sciatic nerve crush from (***A***) with linear regression (***C***) showing a lack of correlation between infection efficiency and length of longest axons. ***D***, Paw withdrawal threshold measurements using von Frey test for recovery of mechanical sensitivity in injured foot pad at noted times after sciatic nerve crush. ***E***, Time spent in pain behavior using acetone test for recovery from cold allodynia in uninjured and injured foot pad at noted times after sciatic nerve crush; *p < 0.05. BL, baseline; D, post-operative day. Extended Data Figure 9-1 shows data values of mean and SEM, number of replicates, statistical tests, and values for all comparisons.

We next tested whether the pro-regenerative environment in Tsc2 KO-AAV8 mice was capable of enhancing initial axon growth after sciatic nerve crush. We analyzed axon length in control-AAV8 and Tsc2 KO-AAV8 sciatic nerves 3 d after a crush injury. Similar to genetic deletion of Tsc2, the longest regenerating axons grew a farther distance in Tsc2 KO-AAV8 nerve compared to control nerve (Fig. 9*A*,*B*), with the length of axon regeneration at 3 d post-injury being similar in Tsc2 cKO and Tsc2 KO-AAV8 mice (compare Figs. 1*D*, 9*B*). Unlike expression of RATFs and macrophage density, increased infection efficiency did not correlate with increased axon length in Tsc2 KO-AAV8 (Fig. 9*C*), suggesting that mTORC1 activation in a relatively low number of neurons is sufficient to observe enhanced axon growth at 3 d post-injury.

10.1523/ENEURO.0168-19.2019.f9-1 Extended Data Figure 9-1 Data values, biological replicates, and statistics supporting Figure 9. Download Figure 9-1, XLSX file.

We next assessed whether the enhanced initial axon regeneration mediated by adult deletion of Tsc2 improved functional recovery. The efficiency of Tsc2 deletion was unchanged after this 10-week experiment compared to the 3.5-week axon regeneration experiment (3.5 weeks 36.8 ± 5.3% Tsc2-positive neurons vs 10 weeks 33.7 ± 5.2% Tsc2-positive neurons). After a sciatic nerve crush, sensitivity to a mechanical stimulus in the foot pad is reduced from loss of innervating axons, which resolves over several weeks as injured axons re-innervate target tissue. Adult deletion of Tsc2 did not affect the course of recovery of mechanical sensitivity (Fig. 9*D*). Specifically, mice lost mechanical sensitivity initially after sciatic nerve crush with control-AAV8 and Tsc2 cKO-AAV8 mice recovering at the same rate.

In our previous study, we observed that Tsc2 deletion in nociceptors reduced cold allodynia in a chronic pain model (Carlin et al., 2018). The region adjacent to the denervated area of the foot after sciatic nerve crush becomes hypersensitive to innocuous cold stimulation. To test whether Tsc2 deletion affects pain behavior in a sciatic nerve crush model, we performed an acetone test weekly after injury to control-AAV8 and Tsc2 cKO-AAV8 mice. Cold allodynia was noted in both groups within one week after nerve crush, and it resolved at four weeks post-injury in both control-AAV8 and Tsc2 KO-AAV8 mice (Fig. 9*E*). Interestingly, we observed an increase injury-induced cold allodynia in Tsc2 KO-AAV8 mice at three weeks post-crush compared to control-AAV8 mice, but not in the acute or post-resolution phases (Fig. 9*E*). Adult deletion of Tsc2 may increase injury-induced hypersensitivity, which was not observed in Tsc2 cKO (Carlin et al., 2018), suggesting that perinatal and adult activation of mTORC1 signaling may have differing effects on some pain responses. Together, these results with adult deletion of Tsc2 yield further support to the notion that mTORC1 activation in DRG neurons provides an initial but unsustained enhancement of axon growth after nerve injury.

## Discussion

In the current study, we assessed the role mTORC1 activation via Tsc2 deletion in promoting axon growth in peripheral nociceptive neurons after nerve injury. As this signaling pathway is a well-known regulator of cellular metabolism and protein translation (Saxton and Sabatini, 2017), we hypothesized that increased signaling would result in an increased rate of regenerative axon growth. In support of our previous findings (Abe et al., 2010), we found that constitutive mTORC1 activation was able to enhance axon growth in nociceptive neurons, but only within 2 d after nerve injury, at which point the elongation rate of control axons was able to catch up. Gene expression changes in uninjured Tsc2-deleted neurons, including upregulated expression of RATFs known to be both necessary and sufficient for promoting axon growth, are likely to cause the enhanced early axon regeneration. Consistent with the similar elongation rates of control and Tsc2 cKO axons after the second day post-injury, Tsc2 deletion did not induce an additive effect on injury-induced upregulation of RATFs or their targets. In addition to changes in neuronal gene expression, neuronal Tsc2 deletion increased macrophage density in uninjured DRG as well as activated macrophages and satellite glial cells in a similar manner as nerve injury. While mTORC1 inhibition studies suggest that neuronal gene expression changes are the main contributors to enhanced early regeneration in Tsc2 cKO mice, some non-neuronal contribution is also likely. We uncover a surprising amount of overlap between the neuronal and non-neuronal pro-regenerative programs of uninjured Tsc2 cKO and injured control DRG. However, despite the extensive overlap in the pro-regenerative programs of Tsc2 deletion and nerve injury, Tsc2 deletion did not fully induced an injury-conditioned state in cultured neurons suggesting that mTORC1 activation induces a partial pro-regenerative program.

mTORC1 signaling promotes axon growth in multiple types of neurons in both the central and PNSs (Park et al., 2008; Abe et al., 2010; Liu et al., 2010). An important distinction between the PNS and CNS is the intrinsic regenerative growth capacity of adult peripheral axons compared to the lack of growth competence of adult central axons. Specifically, manipulation of signaling pathways or transcription factors such as PI 3-kinase, mTORC1, B-Raf, Stat3, c-Myc, Sox11, Klfs, and others is required for axon regeneration after an optic nerve crush, with manipulation of multiple pathways providing additive or synergistic effects on axon elongation (Park et al., 2008; Moore et al., 2009; Smith et al., 2009; Sun et al., 2011; O'Donovan et al., 2014; Belin et al., 2015; Benowitz et al., 2017; Norsworthy et al., 2017). Similar experiments assessing manipulation of multiple pathways after peripheral nerve injury have been less common. A study in mice with AAV-mediated co-deletion of both Pten and Socs3 showed marginal improvements in functional recovery and 3-d regeneration after a sciatic nerve crush, although relatively low infection efficiency limits the conclusions that can be drawn on the true effect size of simultaneous dysregulation of these pathways (Gallaher and Steward, 2018). Based on the optic nerve studies, it is likely that altering multiple non-overlapping pathways will be required to induce robust increases in axon elongation. Our analyses identified altered expression or activity of many pro-regenerative transcription factors as a result of Tsc2 deletion, however many of these changes were more robust in injured control neurons. Tsc2 deletion did not increase the elongation rate in injury-conditioned axons, which suggests that mTORC1 activity is not rate limiting in peripheral sensory axon growth or that these processes occur independently of Tsc2 or both. Additionally, negative regulators of growth may also be induced by mTORC1 activation that act as a brake on long-term axon growth enhancement. For example, our RNA-seq analysis indicated that the effect of Tsc2 deletion on expression of Rest, C/ebpd, and Smad2 target genes were inconsistent with injured control neurons. Similarly, the mTORC1 downstream effector S6K1 was recently shown to negatively regulate axon regeneration in cortical neurons by suppressing PI 3-kinase activation through a mechanism that may also function in DRG neurons (Melemedjian et al., 2013; Al-Ali et al., 2017). Tsc2 deletion in DRG neurons did not induce a complete regenerative program, which may result from mechanistic differences in activation between injury and Tsc2 deletion suggested by their respective sensitivities to rapamycin or from cell type-specific responses to Tsc2 deletion. A better mechanistic understanding of the functions of regeneration-associated signaling pathways and transcription factors and their effects on specific neuronal subtypes may uncover productive combinations of factors to manipulate for induction of synergistic regenerative axon growth in the periphery similar to what has been observed in the optic nerve.

Our study focuses on the neuronal Tsc2/mTORC1 signaling axis in the cell body, however mTORC1 signaling is also active in both naïve and injured axons. An active form of mTORC1 is present in uninjured A-fiber sensory axons, and plantar administration of rapamycin inhibits responses to some painful stimuli (Jiménez-Díaz et al., 2008). Nerve injury upregulates local translation of mTOR protein in injured axons, which increases Stat3 signaling and promotes proprioceptor survival (Terenzio et al., 2018). A requirement for mTORC1 signaling in growth cone dynamics has also been demonstrated in a number of neuronal cell types including DRG neurons (Verma et al., 2005; Nie et al., 2010; Poulopoulos et al., 2019). Future studies will be needed to assess whether Tsc2 deletion is sufficient to activate the axonal pool of mTORC1, and furthermore if increased axonal mTORC1 signaling has a different effect on axon elongation than whole neuron Tsc2 deletion.

The effects of non-neuronal cells on axon regeneration is an emerging field with limited data characterizing the roles for different cell types. However, it is of no surprise that a complex long-term process such as axon regeneration requires glial and immune support. In addition to gene expression changes in neurons, we observed macrophage and satellite glial cell activation as a result of nociceptor deletion of Tsc2. A role for macrophage activation in inducing a pro-regenerative state in DRG as well as myelin clearance in the sciatic nerve has been demonstrated (for review, see Zigmond and Echevarria, 2019). Specifically, Ccr2-mediated recruitment and activation of M2 macrophages via Ccl2 signaling is both necessary and sufficient for axon regeneration via activation of Stat3 (Kwon et al., 2015; Niemi et al., 2016). Our data show that both M1 and M2 macrophages were activated in DRG by nerve injury or nociceptor deletion of Tsc2 with accompanying activation of Stat3. This macrophage activation may be accomplished via observed upregulation of *Ccl8* expression or potentially through post-transcriptional upregulation of other Ccr2 ligands. Increased expression of a number of chemokines including *Ccl7* and *Ccl8* was noted in a recent microarray-based gene expression analysis after nerve injury (Cobos et al., 2018); however, this study analyzed whole DRG lysates, precluding determination of the cellular source of expression changes. As *Ccl2* expression was unaffected by Tsc2 deletion, it is possible that macrophage activation was also incomplete as noted by the unchanged expression levels of *CD163* in Tsc2 cKO DRG. Incomplete activation of the M2 phenotype may partially explain the incomplete conditioning response of uninjured Tsc2 cKO neurons *in vitro*.

Peripheral axons are relatively adept at axon regeneration, suggesting that mTORC1 activation would not provide a significant therapeutic benefit after a nerve crush injury based on our results. However, it will be important to assess whether acute activation of mTORC1 signaling can improve functional recovery of sensory neurons after spinal cord injury or nerve repair surgery where initiation of axon regeneration is less efficient. A conditioning sciatic nerve injury can promote axon regeneration after spinal cord injury (Neumann and Woolf, 1999). Similarly, conditioning insults such as electrical stimulation or capsaicin treatment can induce pro-regenerative gene expression in DRG neurons improving axon outgrowth, which can improve functional recovery after a nerve repair (Frey et al., 2018; Senger et al., 2018, 2019; Poitras et al., 2019). As mTORC1 activation in sensory neurons induces pro-regenerative gene expression, short-term activation of the pathway after dorsal column injury or before nerve repair surgery may be sufficient to produce therapeutically relevant benefits in these severe cases.

## Synthesis

Reviewing Editor: Mike Fainzilber, Weizmann Institute of Science Decisions are customarily a result of the Reviewing Editor and the peer reviewers coming together and discussing their recommendations until a consensus is reached. When revisions are invited, a fact-based synthesis statement explaining their decision and outlining what is needed to prepare a revision will be listed below. The following reviewer(s) agreed to reveal their identity: NONE. Note: If this manuscript was transferred from JNeurosci and a decision was made to accept the manuscript without peer review, a brief statement to this effect will instead be what is listed below.

I think this manuscript is much improved and the open mechanistic issues raised in the J Neurosci review are less of a concern for eNeuro. The data are overall solid and finding clear. I appreciate the authors' response to reviewers and the reorganization of the manuscript makes it a much easier read. The apparent shift of the Tsc2 deleted nociceptive neurons into an ‘injury conditioned-like’ state, but lack of sustained differences in regeneration, including function, should be of interest to eNeuro readers and the field. The non-cell autonomous effects on macrophages and schwann/satellite cells are intriguing and will need subsequent work to sort out differences with published work; but this should be published so that the field can help resolve the issue.

There are a few remaining issues that all could be addressed with text changes:

1. The authors' rebuttal states “Initially, we quantified the percentage of CGRP- and IB4-positive neurons that co-express Rosa-GFP after AAV8 injection and observed no differences in the infection efficiency between those subtypes and all DRG neurons”. I did not see where this is mentioned in the manuscript, and I think this is an important point in evaluating the results (sorry if I missed this).

2. I think the authors should provide a rationale for use of IB4 on page 18 where it is introduced (i.e., for Fig 5). The sentence on page 21 describing the IB4 use for detection of non-peptidergic nociceptors would be useful in this context. This may have been missed during reorganization of the results section?

3. On page 16 sentence beginning “To assess whether RAFT ...” - ‘affect’ should be ‘affected’.

## Author Response

Response to Reviewer's comments:

This manuscript is much improved and the open mechanistic issues raised in the J Neurosci review are less of a concern for eNeuro. The data are overall solid and finding clear. The reorganization of the manuscript makes it a much easier read. The apparent shift of the Tsc2 deleted nociceptive neurons into an ‘injury conditioned-like’ state, but lack of sustained differences in regeneration, including function, should be of interest to eNeuro readers and the field. The non-cell autonomous effects on macrophages and schwann/satellite cells are intriguing and will need subsequent work to sort out differences with published work; but this should be published so that the field can help resolve the issue.

We appreciate the reviewers' critical reading of our revised manuscript and thank them for their kind words. We certainly agree that here is much to be resolved beyond this manuscript, especially the non-cell autonomous effects. Our findings should provide critical input to future studies form the field.

There are a few remaining issues that all could be addressed with text changes:

1. The authors' rebuttal states “Initially, we quantified the percentage of CGRP- and IB4-positive neurons that co-express Rosa-GFP after AAV8 injection and observed no differences in the infection efficiency between those subtypes and all DRG neurons”. I did not see where this is mentioned in the manuscript, and I think this is an important point in evaluating the results (sorry if I missed this).

We thank the reviewers for bringing up this point. As AAVs are increasingly used it is important to know what cells are being infected. We added several sentences in the text on p. 23 related to Figure 8 for N=5 Tsc2 KO-AAV8 infection efficiency of all neurons, CGRP-positive and IB4-positive neurons using a GFP reporter. We did not observe any difference in viral infection by subtype and the text now reflects that point.

2. I think the authors should provide a rationale for use of IB4 on page 18 where it is introduced (i.e., for Fig 5). The sentence on page 21 describing the IB4 use for detection of non-peptidergic nociceptors would be useful in this context. This may have been missed during reorganization of the results section?

We agree and have moved the rationale for use of IB4 from p. 21 to p. 18, which relates to the cultured neurons experiments described in Figure 5.

3. On page 16 sentence beginning “To assess whether RAFT ...” - ‘affect’ should be ‘affected’.

Thank your catching this error, which has been corrected.
